# Genetic Instability Due to Spindle Anomalies Visualized in Mutants of *Dictyostelium*

**DOI:** 10.3390/cells10092240

**Published:** 2021-08-29

**Authors:** Mary Ecke, Jana Prassler, Günther Gerisch

**Affiliations:** Cell Dynamics Group, Max Planck Institute of Biochemistry, am Klopferspitz 18, D-82152 Martinsried, Germany; ecke@biochem.mpg.de (M.E.); prassler@biochem.mpg.de (J.P.)

**Keywords:** centrosomes, chromosome segregation, *Dictyostelium*, genetic instability, mitosis, multinucleate cells

## Abstract

Aberrant centrosome activities in mutants of *Dictyostelium discoideum* result in anomalies of mitotic spindles that affect the reliability of chromosome segregation. Genetic instabilities caused by these deficiencies are tolerated in multinucleate cells, which can be produced by electric-pulse induced cell fusion as a source for aberrations in the mitotic apparatus of the mutant cells. Dual-color fluorescence labeling of the microtubule system and the chromosomes in live cells revealed the variability of spindle arrangements, of centrosome-nuclear interactions, and of chromosome segregation in the atypical mitoses observed.

## 1. Introduction

In *Dictyostelium* as in many other cells, reliable chromosome segregation during mitosis depends on the association of one centrosome with the nucleus, the division of this centrosome, and the formation of an elongating spindle between the daughter centrosomes. 

The notion that aberrations in centrosome function result in unreliable chromosome segregation, causing genetic instability, has a long history. In particular, Theodor Bovery emphasized in 1914 that tumor cells may be derived from genetic alterations that were caused by the impaired function of centrosomes [1].

To visualize spindle aberrations caused by impaired centrosome–nuclear interactions, we used mutants of *Dictyostelium* in which these interactions were destabilized, and labeled the cells with two fluorescence markers, one for the microtubule system including the centrosomes, the other for the chromosomes. The aberrations were observed in large multinucleate cells in which nuclei with aberrant chromosome sets could persist.

*Dictyostelium* cells perform a semi-closed mitosis: at the G2/M interphase, tubulin is translocated into the nucleus, assembling there into the spindle [2]. To induce spindle formation, the centrosome translocates from its position at the outer nuclear membrane into the nucleus and divides there; the two daughter centrosomes build the two poles of the spindle [3]. 

During elongation of the spindle within the cytoplasmic space, the chromosomes are segregated, remaining enveloped by the nuclear membrane. During anaphase, the centrosomes reassume their position on the outside of the nucleus, forming aster microtubules that contact the plasma membrane at the two poles of the cell [4]. Finally, the spindle disrupts in the midzone and a cleavage furrow ingresses.

Several types of mutations destabilize the association of the centrosome with the nucleus, resulting in atypical spindle formation and aberrations in chromosome segregation. Among the proteins affected by these mutations are actin-binding proteins such as Aip1 (actin-interacting protein 1) involved in actin-filament depolymerization or the actin filament anti-parallel bundling protein cortexillin [5]. In mutants lacking one of these proteins, variable numbers of centrosomes can associate with one nucleus and, as shown in Aip1- null cells, the centrosomes can reversibly detach from the nucleus.

Here we provide an overview of aberrant mitoses observed in three mutants deficient in regulatory proteins of the actin system: in Septase-null, RacB-null, and RacE-null mutants. Most of the examples are taken from mitoses in Septase-null cells. Septase-null cells tend to become multinucleate because of inefficient cytokinesis [6]. Septase (SepA) is a serine/threonine kinase, homologous to Cdc7 of *Schizosaccharomyces pombe*. In *Dictyostelium* cells, Septase phosphorylates Scar/WAVE and in that way controls the lifetime of pseudopods [7]. RacB has been identified as a regulator of actin polymerization in response to chemoattractant [8]. A role for RacB in mitosis was suggested by cells expressing constitutively active RacB. These cells had nuclei of heterogeneous sizes, both larger and smaller than in parental cells [9]. RacE was discovered in a screen for genes required for cytokinesis; RacE-null cells are impaired in cytokinesis when cultivated in suspension [10]. It regulates the three diaphanous-related formins A, E, and H in *Dictyostelium* cells, and is required for maintaining the rigidity of the cell cortex [11]. The role of Rho-GTPases in the cytokinesis of *Dictyostelium* cells is reviewed in [12].

## 2. Materials and Methods

### 2.1. Cell Strains

Green and red fluorescent proteins (GFP and RFP, respectively) were expressed in the AX2-214 strain of *Dictyostelium discoideum*, here designated as “wild-type”, and in three mutants derived from this strain: RacB-null [8], RacE-null [10,11], and Septase-null [6] (Table 1).

### 2.2. Culture Conditions and Sample Preparation for Confocal Microscopy

Cells were cultivated in nutrient medium as described by Malchow et al. [20], supplemented with 10 µg/mL of Blasticidin S (Gibco, Life Technologies Corporation, Grand Island, NY, USA), 10 µg/ml of Geneticin (Sigma-Aldrich, St. Louis, MO, USA), and/or of 33 µg/mL of Hygromycin B (EMD Millipore Corp., Billerica, MA, USA) in plastic Petri dishes at 21 ± 2 °C.

Unfused cells were incubated overnight in Loflow medium (ForMedium Ltd., Norfolk, UK) or kept at 4 °C to increase the rate of mitotic stages. The following day, cells were brought to room temperature and transferred onto HCl-cleaned cover-glass bottom dishes (FluoroDish, WPI INC., Sarasota, FL, USA) with Loflow medium. Imaging began after 1 to 3 h.

Large multinucleate cells of RacB-null and RacE-null mutants were produced by electric-pulse-induced fusion in 17 mM phosphate buffer, pH 6.0, as described by Gerhardt et al. [21]. After the fusion, cells were incubated up to 6 h in Loflow medium (ForMedium Ltd., Norfolk, UK) before imaging to increase the rate of mitosis.

Multinucleate Septase null-cells were rinsed off the Petri dish with 17 mM phosphate buffer, pH 6.0, and transferred to an HCl-cleaned cover-glass bottom dish. A 3-hour incubation time in the phosphate buffer was followed by an incubation in Loflow medium for 4 to 6 h to synchronize mitotic entry.

Where indicated in the figure legends, the cells were overlaid by a thin agarose sheet [22], since slight compression facilitated confocal imaging.

### 2.3. Confocal Image Acquisition and Data Processing

Confocal images were acquired on a Zeiss LSM 780 microscope equipped with a Plan-Apochromat 63×/NA 1.46 oil immersion or a Plan-Apochromat 40×/NA 1.2 water objective (Zeiss AG, Oberkochen, Germany). The images were processed using the image-processing package Fiji (http://Fiji.sc/Fiji (accessed on 26 July 2021)) developed by Schindelin et al. [23] on the basis of ImageJ (http://imagej.nih.gov/ij (accessed on 26 July 2021)). In most figures, we show average projections of a series of confocal planes.

## 3. Results

### 3.1. Normal Mitosis in D. discoideum

**Figure 1:**
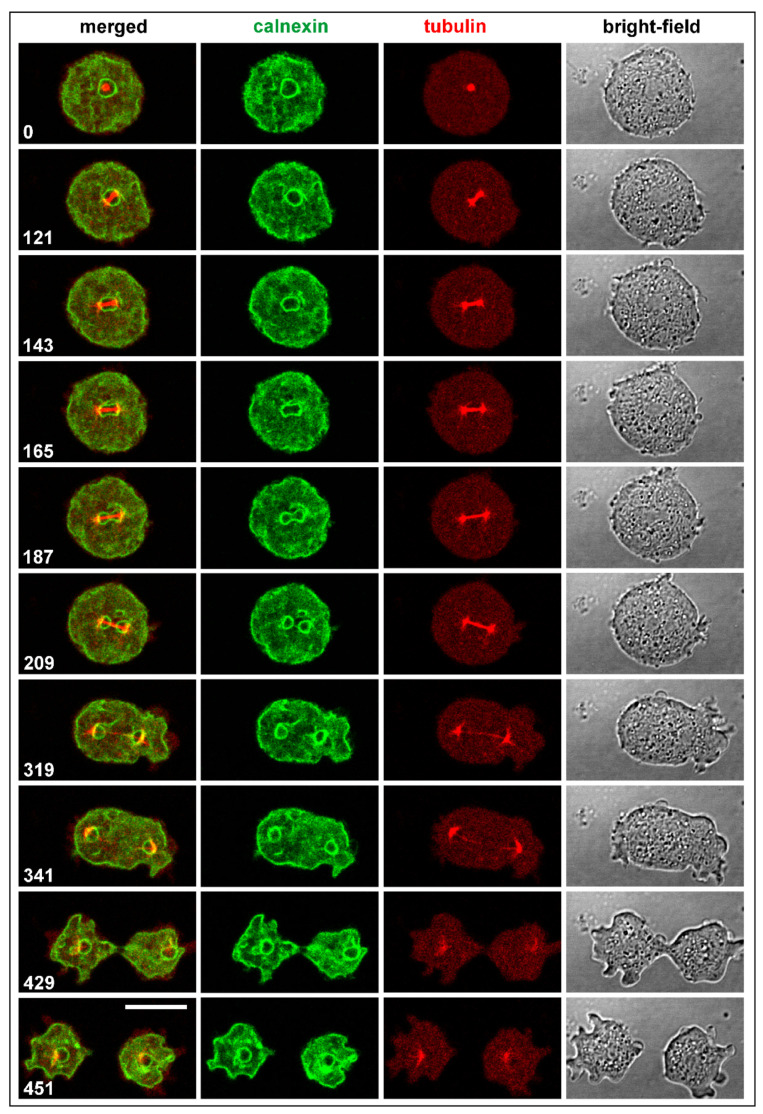
Normal mitosis in *D. discoideum* showing spindle formation and elongation relative to the outer nuclear membrane. The cell expressed GFP-calnexin to label the endoplasmic reticulum that comprises the outer nuclear membrane, and mRFP-α-tubulin to visualize the mitotic apparatus. Panels show from left to right: merged fluorescence images displaying the Calnexin label in green and the α-tubulin label in red; the Calnexin label separately; the α-tubulin label; and confocal bright-field images of the dividing cell. This sequence is also shown in Video S1. Time is indicated in seconds. Bar, 10 µm.

As revealed by labeling the endoplasmic reticulum, including the outer nuclear membrane, with Calnexin-GFP, the spindle remained enveloped at the beginning of its elongation by the outer nuclear membrane, which was disrupted prior to disassembly of the spindle at the midzone (Figure 1 and Video S1).

**Figure 2:**
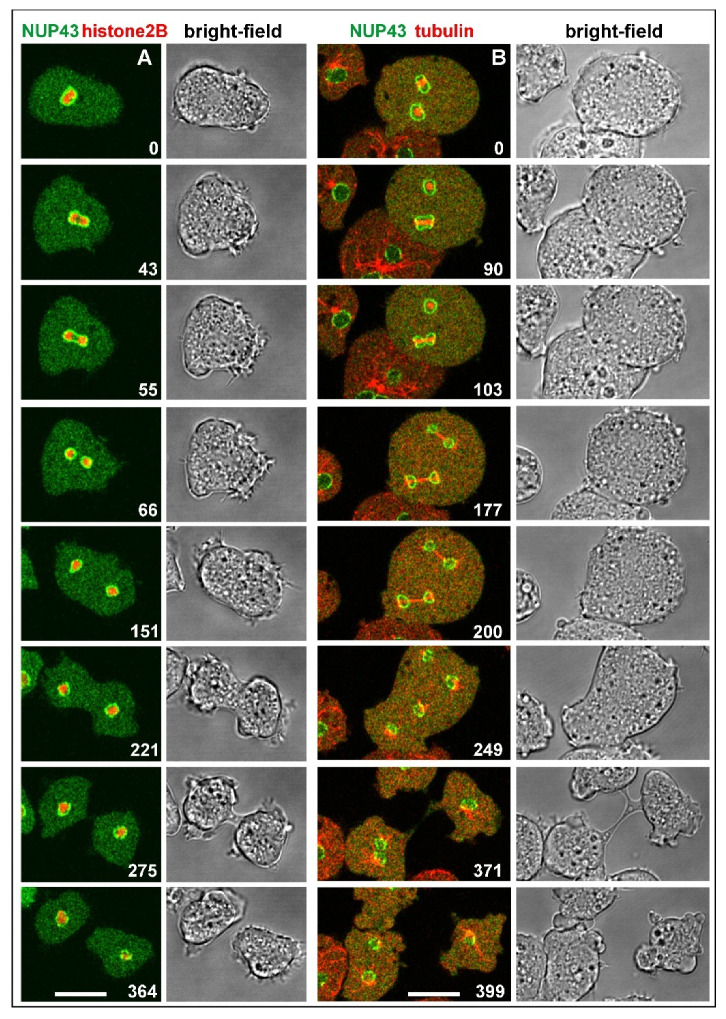
Normal mitosis of cells showing persistence of the inner nuclear membrane. (**A**), a cell expressing NUP43-GFP to label the nuclear membrane (green) and mRFP-histone2B to visualize the chromosomes (red), which remain enveloped thoughout mitosis. (**B**), a binucleate cell expressing NUP43-GFP (green), and mRFP-α-tubulin (red) to label centrosomes and spindles. Ingression of the cleavage furrow commences after disruption of the spindle (249-s to 371-s frames). The cell divides into three daughter cells, two uninucleate ones, and a binucleate one. An interphase cell on the left shows, for comparison, the centrosome attached to the nuclear membrane, microtubules in the cytoplasmic space but no tubulin within the nucleus. The cells were slightly compressed by agar overlay. Time is indicated in seconds. Bars, 10 µm.

The nuclei of *Dictyostelium* are profusely decorated with nuclear pore complexes, which span the entire nuclear membrane [24]. To demonstrate continued integration of the pore complexes, implying integrity of the inner nuclear membrane during mitosis, we have used a GFP-tagged nuclear pore protein, NUP43, in combination with either mRFP-α-tubulin to label the mitotic apparatus, or with mRFP-histone2B to label the chromosomes. The chromosomes remain enclosed by the nuclear envelope during all stages of mitosis, while the spindle elongates outside of this boundary (Figure 2).

### 3.2. Spindle Anomalies Caused by Disturbed Centrosome Activities

**Figure 3:**
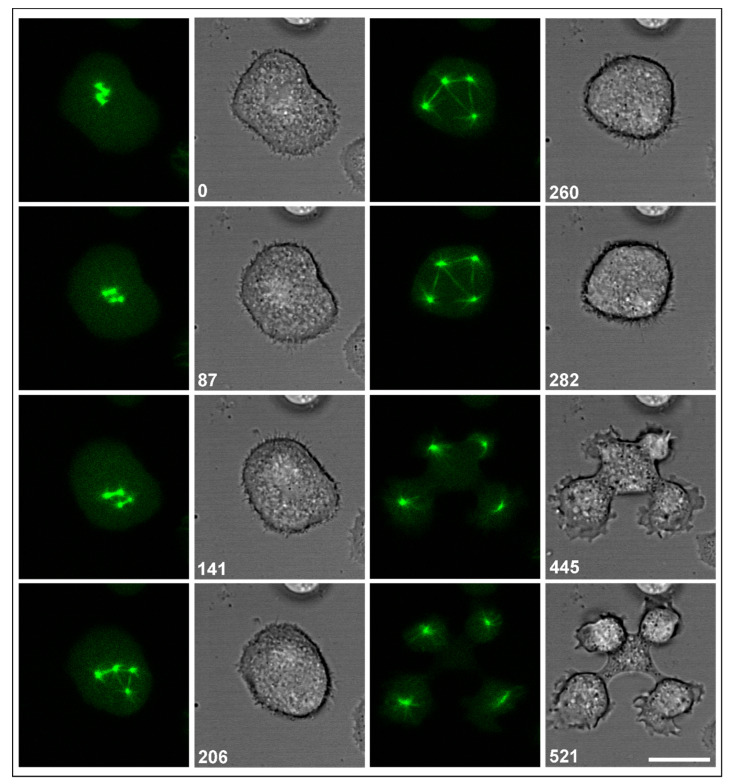
Multipolar centrosomes connected by spindles. The RacB-null cell expressed GFP-α-tubulin (**left** panels), showing the centrosomes (0-s and 87-s frames) and elongating spindles (141-s to 282-s frames). In parallel, cell shapes are displayed in confocal bright-field images (**right** panels). The cell divided regularly into four daughter cells (445-s and 521-s frames). Time is indicated in seconds. Bar, 10 µm.

In Figure 3, a system of multiple interconnected centrosomes is shown, generated in a RacB-null cell by two metaphase complexes in close apposition. When the spindles elongated, three of the four centrosomes remained connected by multiple spindles, which implies that each of these centrosomes became the pole of at least two spindles. Despite these aberrations in the arrangement of spindles, the cell regularly divided into four daughter cells, forming cleavage furrows not only at the sites where centrosomes were connected by a spindle, but also at a space where no spindle connected the centrosomes.

**Figure 4:**
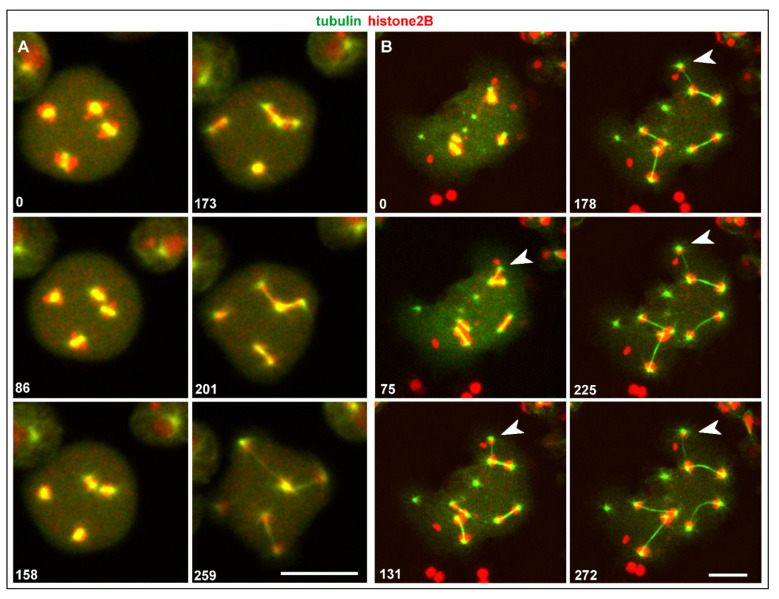
Centrosome fusion followed by unequal sets of chromosomes translocated by two spindles. Cells expressed GFP-α-tubulin to visualize the mitotic apparatus (green) and RFP-histone2B to label the chromosomes (red). (**A**), centrosome fusion in a tetra-nucleate cell of a RacE-null mutant. Up to the metaphase stage, four separate mitotic complexes persisted (0-s to 158-s frames). Subsequently, two complexes joined each other by the fusion of two adjacent centrosomes (173-s and 201-s frames). As a result, the two elongating spindles formed by these complexes had one centrosome in common (201-s to 259-s frames). (**B**), in a Septase-null cell, two spindles arose from three centrosomes associated with one large nucleus. Only a small amount of histone-labeled material became translocated to the upper centrosome (arrowhead), indicating that an incomplete set of chromosomes was translocated to this pole. The cell is also shown in Video S2. Time is indicated in seconds. Bars, 10 µm.

The RacE-null cell shown in Figure 4A contained four mitotic complexes, two of which came very close to each other. In this case, two centrosomes (one of each complex) fused, such that two spindles formed that had one pole in common.

### 3.3. Spindle Anomalies Resulting in Aberrant Chromosome Segregation

**Figure 5:**
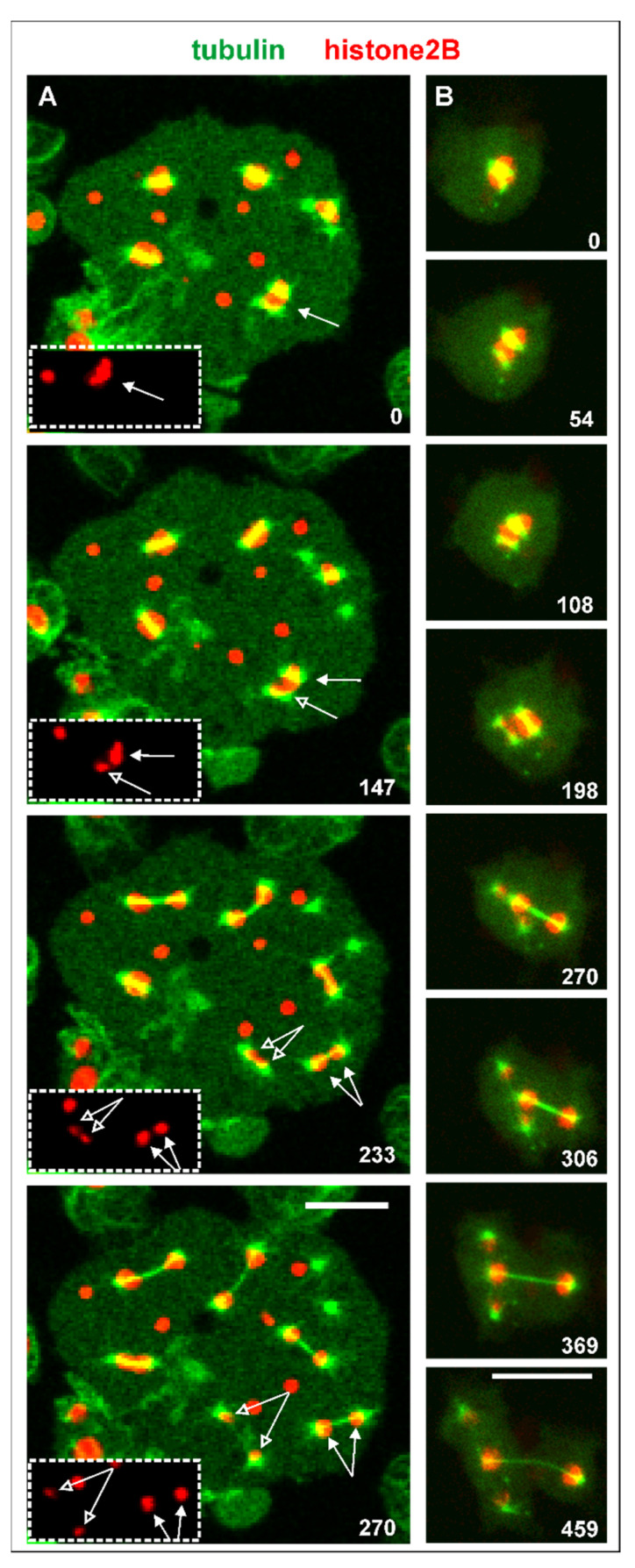
Mitotic complexes with incomplete sets of chromosomes. Septase-null cells expressed GFP-α-tubulin as a label Figure 2. B for the chromosomes (red). (**A**), mitosis in a multinucleate cell. Two mitotic complexes distinguished by different amounts of histone-labeled material are indicated by closed and open arrows. In the boxed area, these two complexes are displayed in the histone2B channel only. The cell was compressed by agar overlay. (**B**), four centrosomes attached to a large nucleus, forming two spindles that translocated different sets of chromosomes to their poles. The cell is also shown in Video S3. Time is indicated in seconds. Bars, 10 µm.

In the case of three centrosomes associated with one nucleus that is shown in Figure 4B and Video S2, one of the centrosomes acquired fewer chromosomes than the other two (arrowhead), indicating genetic instability as an outcome of this configuration. This case suggests that spindle elongation and chromosome segregation do not require a complete set of chromosomes. Evidence for that is provided by the multinucleate cell shown in Figure 5A. This cell contained isolated chromatin clusters, single centrosomes, and six mitotic complexes. One of these complexes became distinguished by a very small mass of chromatin, probably one or two chromosomes translocated to each spindle pole (arrows in Figure 5A).

Another example of unequal chromosome segregation is displayed in Figure 5B and Video S3. Four centrosomes attached to one nucleus to form two spindles that transported different amounts of histone-labeled chromosomes. Remarkably, these two spindles differed also in thickness, indicating different numbers of spindle microtubules generated by the two pairs of centrosomes.

### 3.4. Single Centrosomes Dividing without Forming a Spindle

**Figure 6:**
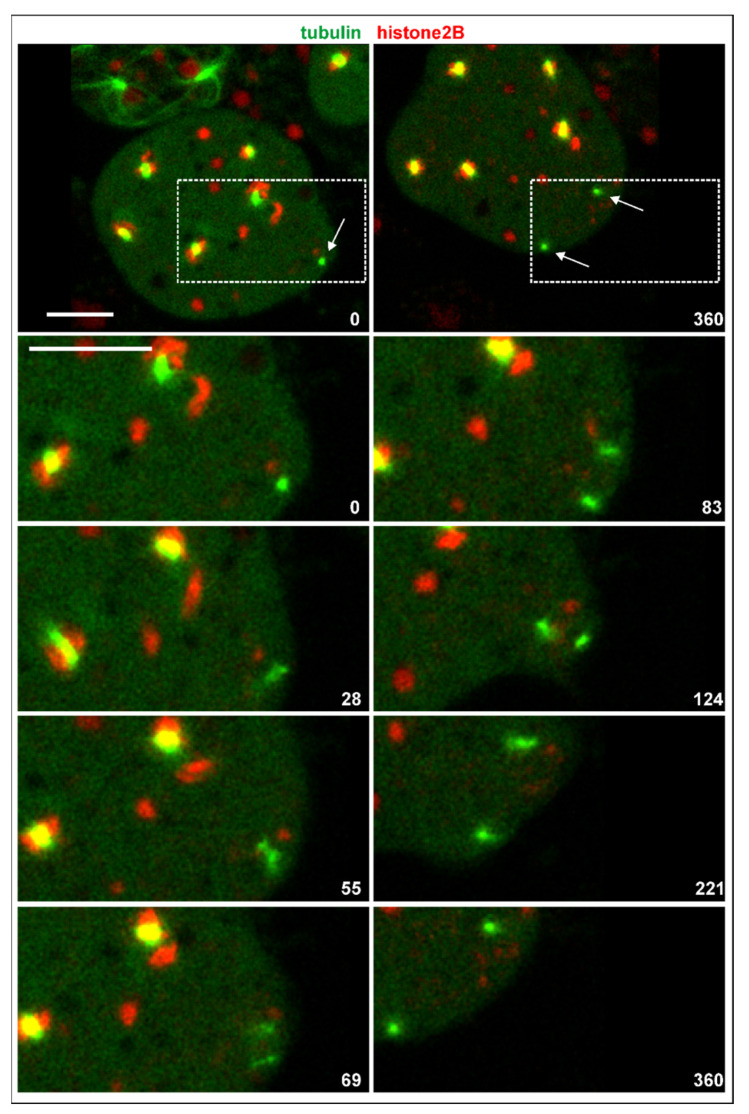
Division of an isolated centrosome. The RacE-null cell expressed GFP-α-tubulin to label the mitotic apparatus including the centrosomes (green) and mRFP-histone2B (chromatin structures red). The two upper panels show the entire cell at the beginning and 6 min period. Division of a centrosome is indicated by arrows. The boxed area is magnified in the lower panels to highlight division of the single centrosome with no spindle being formed. The cell was compressed by agar overlay. Time is indicated in seconds. Bars, 10 µm.

In multinucleate cells, centrosomes detached from a nucleus still divided, and they did so in synchrony with nuclear-associated centrosomes. In contrast to the latter, the seven single centrosomes scored did not remain connected by a spindle (Figure 6). In one case, a thin microtubular connection persisted for 3 min between the daughter centrosomes, which appeared to consist of two cross-linked anti-parallel aster microtubules. In any case, the daughter centrosomes formed microtubule asters and were capable of translocating.

### 3.5. Shape Changes of Multicentric Nuclei in Interphase

**Figure 7:**
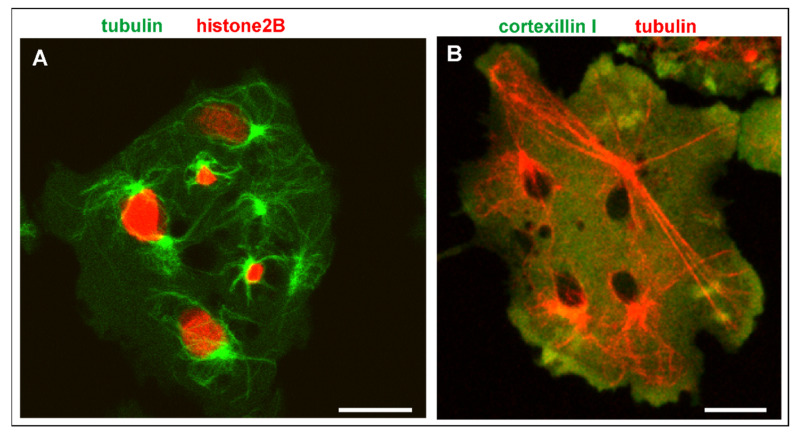
Multinucleate cells in interphase. (**A**), heterogeneity of nuclear sizes in a RacB-null cell. This cell expressed mRFP-histone2B (red) to label the nuclei, and GFP-α-tubulin (green) to visualize nuclear-bound and free centrosomes with their cytoplasmic microtubule systems in the interphase stage. (**B**), a Septase-null cell with extremely long microtubules that connect a centrosome with the cell cortex. The cell expressed mRFP-α-tubulin (red) and GFP-cortexillin I (green). The black areas are the nuclei. The cells were compressed by agar overlay. Bars, 10 µm.

**Figure 8:**
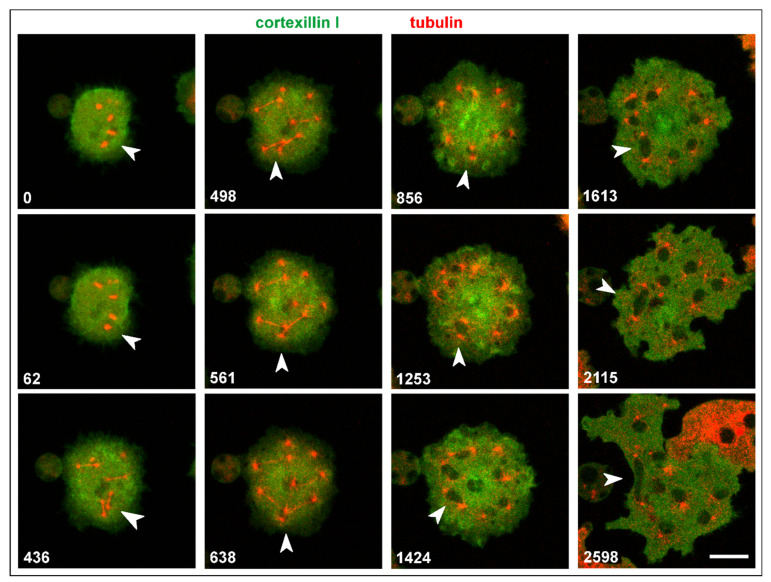
Two adjacent spindles join to form a bicentric nucleus. During mitosis of a multinucleate Septase-null cell, one pole of each of two spindles associated with the same daughter nucleus (arrowheads). The Septase-null cell expressed GFP-cortexillin I (green) and RFP-α-tubulin to label the mitotic apparatus (red), which leaves the nuclei unstained. After mitosis, this nucleus is strongly elongated between the two centrosomes. The cell was compressed by an agar overlay; it is also shown in Supplementary Video S4. Bars, 10 µm.

In mutant interphase cells, the consequences of genetic instability are obvious, although in multinucleate cells, they do not result in lethality. Nuclei of heterogeneous size and isolated centrosomes persist in the interphase stage (Figure 7A). Microtubules connect the centrosomes with the cell cortex and can become extremely long in multinucleate cells (Figure 7B). Figure 8 and Video S4 show how a bicentric nucleus is generated and is moved and deformed in the consecutive interphase by the two attached centrosomes.

**Figure 9:**
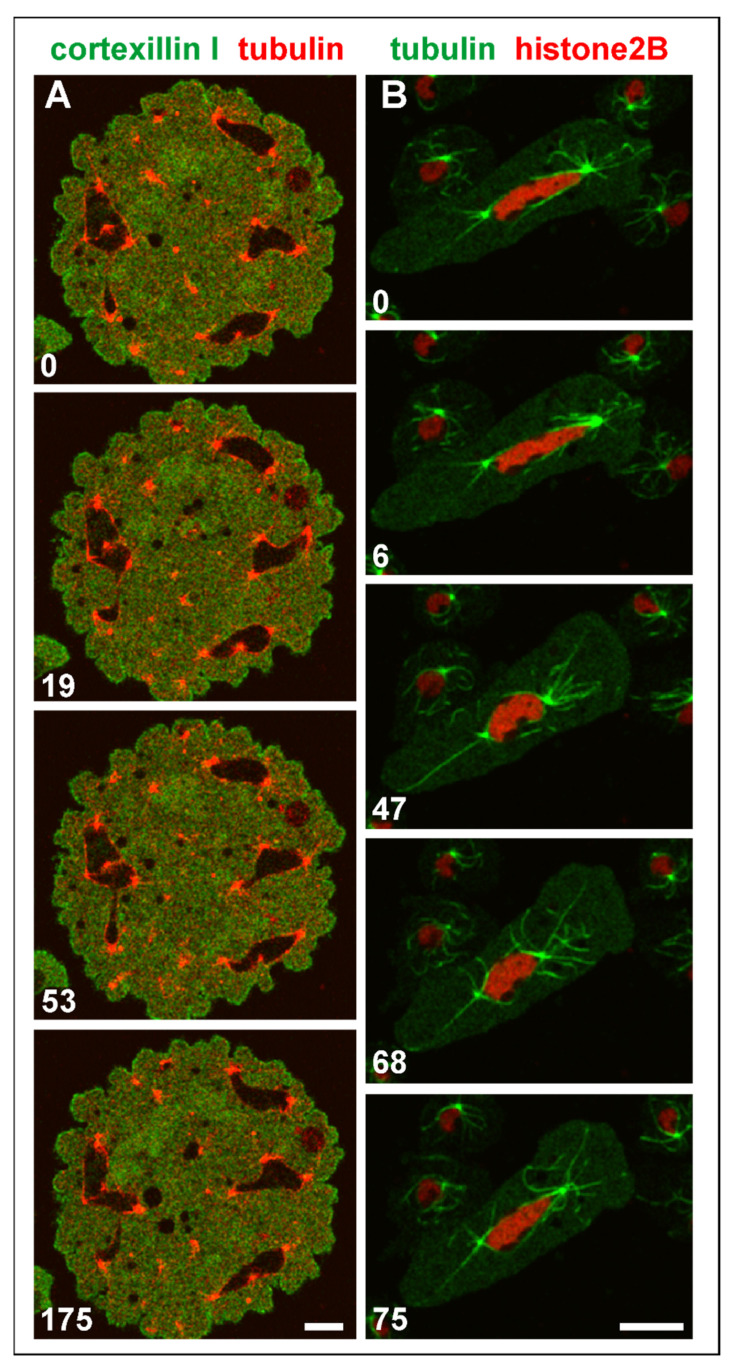
Shape changes of large nuclei with multiple centrosomes. (**A**), a multinucleate Septase-null cell that expressed RFP-α-tubulin to label centrosomes and microtubules (red) and GFP-Cortexillin I (green), which localizes to the cytoplasm such that the nuclei appear as black areas in this interphase cell. The cell is also shown in Video S5. (**B**), elongation of a large nucleus between two centrosomes that were anchored by microtubules to opposite positions of the cell cortex. The RacB-null cell expressed GFP-α-tubulin to label the mitotic apparatus (green), and RFP-histone2B for the nucleus (red). Long microtubules that anchor the centrosomes to the cell cortex are seen in the 47-s, 68-s, and 75-s frames. The cell is also shown in Video S6. The cells in (**A**,**B**) were compressed by an agar overlay. Time is indicated in seconds. Bars, 10 µm.

Both single and attached centrosomes are moved by the bunches of associated microtubules, which can result in heavy deformations of multicentric nuclei (Figure 9A and Video S5). One of these nuclei is shown in Figure 9B and Video S6: it was extremely stretched between two centrosomes that were connected by microtubules with the cell cortex.

## 4. Discussion

In this report, we have provided examples of spindle anomalies as a basis of unreliable chromosome segregation in mitosis. Previous work showed that these anomalies result in aneuploidy and suggested that the affected cells were nonviable [25]. The multinucleate cells used here appear to balance deficiencies in single nuclei by the multitude of other nuclei and their poly- or aneuploidy, thereby tolerating genetic instabilities. Live-imaging was applied to the multinucleate cells that either formed spontaneously by deficient cytokinesis in mutant cells or were produced by electric-pulse-induced cell fusion [26].

The spindle anomalies are caused by aberrant centrosome activities. The observed aberrations comprised (1) detachment of centrosomes from the nucleus, (2) association of multiple centrosomes with one nucleus, and (3) centrosome fusion.

(1) Centrosome–nuclear connections are impaired in mutants deficient in dynein activity [25], in cells depleted of the inner nuclear membrane protein Sun-1 [13], and in mutants lacking a kinesin, Kif9 [27]. This motor protein has a membrane-binding domain and is localized to the nuclear envelope beneath the centrosome-attachment site [2]. Kif9 engages microtubules to draw the centrosome to the nucleus.

Centrosomes detached from nuclei are capable of dividing separately, as first reported by Gräf et al. [28] for mutants overexpressing the microtubule-associated protein DdCP224. Normally, a spindle is formed after the centrosome has divided and entered the nucleus [3]. A matter of discussion is whether free centrosomes can form a spindle and whether their division is delayed relative to that of nuclear-associated centrosomes [27,28]. Under our conditions, all the free centrosomes scored divided synchronously with the nuclear-attached centrosomes. The isolated centrosomes formed microtubule asters, but we did not observe a proper spindle between them. These results agree with the findings reported by Leo et al. [27] for Kif9-null cells.

(2) Aberrations in centrosome activity may result in abnormally large nuclei often associated with multiple centrosomes. This is reminiscent of the aneuploid nuclei found in mutant cells with deficiencies in centrosome replication, such as cells impaired in dynein activities [25] or depleted in Sun-1 [13]. Another way of producing a bicentric nucleus is the joining of two spindles, as shown in Figure 8 and Video S4.

(3) When two centrosomes fuse, they can become one common pole of two or three spindles (Figure 3). Multiple spindles thus distribute the chromosomes of a large nucleus to more than two poles (Figure 4B, Video S2 and Figure 5, Video S3).

Finally, we wish to emphasize that the three mutants studied here have been known for their deficiencies in the actin cytoskeleton, rather than in the microtubule system. These results point to a role of the actin system in mitotic centrosome and spindle functions, consistent with the function of actin in other systems, e.g., its role in chromosome congression [29], chromosome alignment [30], and the action of the centrosome in promoting Arp2/3-dependent actin-filament assembly [31].

## 5. Conclusions

The generation of multinucleate cells in combination with the use of mutants in which the regular association of one centrosome per nucleus is destabilized, provides a rich source of anomalies in the structure and function of the mitotic apparatus that result in unreliable chromosome segregation and thus in genetic instability.

## Figures and Tables

**Table 1:** List of strains in this paper.

Strain	GFP Label	RFP Label	References
AX2-214	NUP43-GFP	mRFPM-α-tubulin	[13,14]
AX2-214	Calnexin-GFP	mRFPM-α-tubulin	[14,15]
RacE-null	pDRH-Hyg^R^:GFP- α-tubulin	mRFP1-histone2B	[16,17]
RacB-null	pDRH-Hyg^R^:GFP- α-tubulin	mRFP1-histone2B	[16,17]
Septase-null	GFP-α-tubulin	mRFP1-histone2B	[4,17]
Septase-null	GFP-cortexillin I	pDRH-Hyg^R^:RFP- α-tubulin	[18,19]

## Data Availability

If not contained within the article or Supplementary Material of this publication, data will be available from the corresponding author.

## Supplementary Materials

The following are available online at https://www.mdpi.com/article/10.3390/cells10092240/s1, Video S1: Mitotic division of a wild-type cell expressing GFP-calnexin (green) and mRFP-α-tubulin (red), Video S2: A Septase-null cell with four mitotic complexes expressing GFP- α-tubulin (green) and mRFP-histone2B (red), Video S3: A Septase-null cell showing four centrosomes associated with one nucleus, Video S4: A multinucleate Septase-null cell showing the formation of a bicentric nucleus, Video S5: A Septase-null cell in interphase displaying shape changes among the large nuclei with two or three attached centrosomes, and the movement of free centrosomes, Video S6: A large nucleus stretched between two centrosomes.
