# Supplementary material for: Genetic Instability Due to Spindle Anomalies Visualized in Mutants of Dictyostelium
Source: Cells. 2021 Aug 29;10(9):2240. doi: 10.3390/cells10092240 (PMC8469108; doi:10.3390/cells10092240)
Supplement: Supplementary file 1 [file cells-10-02240-s001.zip › Ecke et al. Genetic Supplementary Video Legends.pdf]

Genetic instability due to spindle anomalies visualized in mutants of *Dictyostelium*

Mary Ecke, Jana Prassler and Günther Gerisch

Supplementary Video Legends

Video S1. Mitotic division of a wild-type cell expressing GFP-calnexin (green) and mRFP-α-tubulin (red). The four panels show from left to right merged fluorescence images, the calnexin channel only, the α-tubulin channel, and confocal bright field images of the entire dividing cell. This video is related to Figure 1.

Video S2. A Septase-null cell with four mitotic complexes expressing GFP- α-tubulin (green) and mRFP-histone2B (red). The upper complex forms two spindles, the upright one translocating only a small amount of histone-labeled material to the centrosome at the top of the cell (arrow). This video is related to Figure 4B.

Video S3. A Septase-null cell showing four centrosomes associated with one nucleus. The cell expressed GFP- α-tubulin (green) and mRFP-histone2B (red). Two spindles were formed, which translocated different amounts of histone-labeled material, implying different sets of chromosomes. This video is related to Figure 5B.

Video S4. A multinucleate Septase-null cell showing the formation of a bicentric nucleus (at the bottom of the cell). The cell expressed GFP-cortexillin I (green), which outlines the nuclei as dark spots, and mRFP-α-tubulin (red). This video is related to Figure 8.

Video S5. A Septase-null cell in interphase displaying shape changes among the large nuclei with two or three attached centrosomes, and the movement of free centrosomes. The cell expressed GFP-cortexillin I (green) outlining the nuclei in black, and mRFP-α-tubulin (red). This video is related to Figure 9A.

Video S6. A large nucleus stretched between two centrosomes. A RacB-null cell expressed GFP- α-tubulin (green) and mRFP-histone2B (red). The centrosomes are connected through microtubules with the cell cortex. This video is related to Figure 9B.
